# Pre- and Post-Operative Nomograms to Predict Recurrence-Free Probability in Korean Men with Clinically Localized Prostate Cancer

**DOI:** 10.1371/journal.pone.0100053

**Published:** 2014-06-17

**Authors:** Minyong Kang, Chang Wook Jeong, Woo Suk Choi, Yong Hyun Park, Sung Yong Cho, Sangchul Lee, Seung Bae Lee, Ja Hyeon Ku, Sung Kyu Hong, Seok-Soo Byun, Hyeon Jeong, Cheol Kwak, Hyeon Hoe Kim, Eunsik Lee, Sang Eun Lee, Seoul National University-Uro-Oncology Group

**Affiliations:** 1 Department of Urology, Seoul National University Hospital, Seoul, Republic of Korea; 2 Department of Urology, Seoul National University Bundang Hospital, Seongnam, Republic of Korea; 3 Department of Urology, Seoul National University Boramae Hospital, Seoul, Republic of Korea; Innsbruck Medical University, Austria

## Abstract

**Objectives:**

Although the incidence of prostate cancer (PCa) is rapidly increasing in Korea, there are few suitable prediction models for disease recurrence after radical prostatectomy (RP). We established pre- and post-operative nomograms estimating biochemical recurrence (BCR)-free probability after RP in Korean men with clinically localized PCa.

**Patients and Methods:**

Our sampling frame included 3,034 consecutive men with clinically localized PCa who underwent RP at our tertiary centers from June 2004 through July 2011. After inappropriate data exclusion, we evaluated 2,867 patients for the development of nomograms. The Cox proportional hazards regression model was used to develop pre- and post-operative nomograms that predict BCR-free probability. Finally, we resampled from our study cohort 200 times to determine the accuracy of our nomograms on internal validation, which were designated with concordance index (c-index) and further represented by calibration plots.

**Results:**

Over a median of 47 months of follow-up, the estimated BCR-free rate was 87.8% (1 year), 83.8% (2 year), and 72.5% (5 year). In the pre-operative model, Prostate-Specific Antigen (PSA), the proportion of positive biopsy cores, clinical T3a and biopsy Gleason score (GS) were independent predictive factors for BCR, while all relevant predictive factors (PSA, extra-prostatic extension, seminal vesicle invasion, lymph node metastasis, surgical margin, and pathologic GS) were associated with BCR in the post-operative model. The c-index representing predictive accuracy was 0.792 (pre-) and 0.821 (post-operative), showing good fit in the calibration plots.

**Conclusions:**

In summary, we developed pre- and post-operative nomograms predicting BCR-free probability after RP in a large Korean cohort with clinically localized PCa. These nomograms will be provided as the mobile application-based SNUH Prostate Cancer Calculator. Our nomograms can determine patients at high risk of disease recurrence after RP who will benefit from adjuvant therapy.

## Introduction

Prostate cancer (PCa) is the most common malignancy among men worldwide, and is a primary leading cause of cancer-associated death in Western men [1]. After radical prostatectomy (RP), the current gold standard of treatment for clinically localized PCa [2], about 30% of patients who underwent RP developed biochemical recurrence (BCR). These are patients who should receive adjuvant therapy with careful surveillance [3], [4]. Pre-operative and post-operative prediction of BCR is important for patient counseling as well as informed decision-making of proper adjuvant therapeutic strategy [5]. In the past, determination of high risk of failure after RP primarily depended upon final pathologic stage [6]. However, some patients with a low pathologic stage of PCa will later develop recurrence, whereas many patients with an advanced pathologic stage of PCa will remain disease-free [7]. Several validated methods, including risk groupings, probability tables, artificial neural network and nomograms have been developed to predict BCR accurately despite the heterogeneity of PCa [8].

Nomograms are statistical tools that graphically represent a mathematical algorithm of the continuous probability of a particular endpoint. Nomograms currently offer the most accurate and discriminating performance for outcome prediction in patients with PCa [9]. Because these models are based on individual patient characteristics, nomograms have limited prediction accuracy when applied in different populations from those where the model was first developed [10]. Inaccurate performance can be more profound when other significant differences exist, such as ethnic and geographic variation [10]. Thus, nomograms based on Western populations may not predict well when directly applied to Asian, especially Korean populations.

Although the incidence of PCa is rapidly increasing in Korea, there are few suitable prediction models optimized to Korean patients. We recently developed a Korean nomogram to predict the pathological stage of clinically localized PCa after RP [11], and further examined its generalizability with a Korean multicenter cohort [12]. Other Korean nomograms for PCa predict particular variables, such as pathologic stage, extra-prostatic extension (EPE), and lymph node metastasis (LNM) [13]–[15]. However, nomograms to estimate oncologic outcomes, such as BCR-free probability after RP, have not been developed for patients with PCa in Korea, desirable to determine adjuvant therapeutic strategy. We therefore developed pre- and post-operative nomograms that are able to predict BCR-free probability at 1, 2 and 5 years after RP in a large Korean cohort with clinically localized PCa.

## Patients and Methods

### Ethics Statement

The Institutional Review Boards (IRBs) of both Seoul National University Hospital (SNUH) and Seoul National University Bundang Hospital (SNUBH) approved this study (Approval number: SNUH, J-1402-102-560; SNUBH, B-1402-240-105). Because this study was carried out retrospectively, the IRBs waived the written informed consent from patients.

### Patient Population

We reviewed the medical records of 3,034 consecutive patients diagnosed with clinical stage T1c–T3a PCa who underwent RP at SNUH and SNUBH from June 2004 through July 2011. One hundred and fifty-six patients who received radiation or hormone therapy before RP and those whose cancer spontaneously regressed were excluded. Eleven other patients who had missing medical records were also excluded. Thus, 2,867 patients became the cohort for the development and validation of nomograms in this study.

### Examination of Clinicopathologic Factors

Preoperative variables consisting of clinical stage, preoperative Prostate-Specific Antigen (PSA) level, biopsy Gleason score (GS) and the proportion of positive biopsy cores (PPC) from biopsy results were reviewed. Postoperative variables consisting of preoperative PSA, GS of final pathologic results, EPE, seminal vesicle invasion (SVI), LNM and surgical margin (SM) status were also reviewed. PPC was defined as the percentage of positive biopsy cores out of total biopsy cores. Clinical and pathological stages were determined by the 7^th^ edition of the American joint committee on cancer (AJCC) staging system. All prostate samples after RP were fixed in 10% neutral buffered formalin and embedded in a paraffin block. After sectioning at 4-mm thickness using a standardized processing protocol, slides were stained with hematoxylin and eosin (H&E) for pathologic examination. Experienced genitourinary pathologists in each hospital examined all specimens with a standardized reporting protocol. All clinicopathological data used in this study are provided as supporting information (Table S1).

### Statistical Analysis

We constructed Cox proportional hazards (PH) regression models using pre- and post-operative variables, and developed final pre- and post-operative nomograms to predict BCR, defined as a serum PSA value of 0.4 ng/ml or greater after RP.

To check the accuracy of our nomograms by concordance index (c-index), we used a resampling method with 200 bootstrap samples. Calibration plots were generated to examine the association between predictive values and actual data. All of the statistical analyses were carried out using the package ‘rms’ in R for Windows, version 3.0.1 (R Foundation for Statistical Computing, Vienna, Austria. http://www.r-project.org/) with the ‘rms’ package. Null hypotheses of no difference were rejected if *p*-values were less than.05, or, equivalently, if the 95% CIs of risk point estimates excluded 1.

## Results

**Figure 1:**
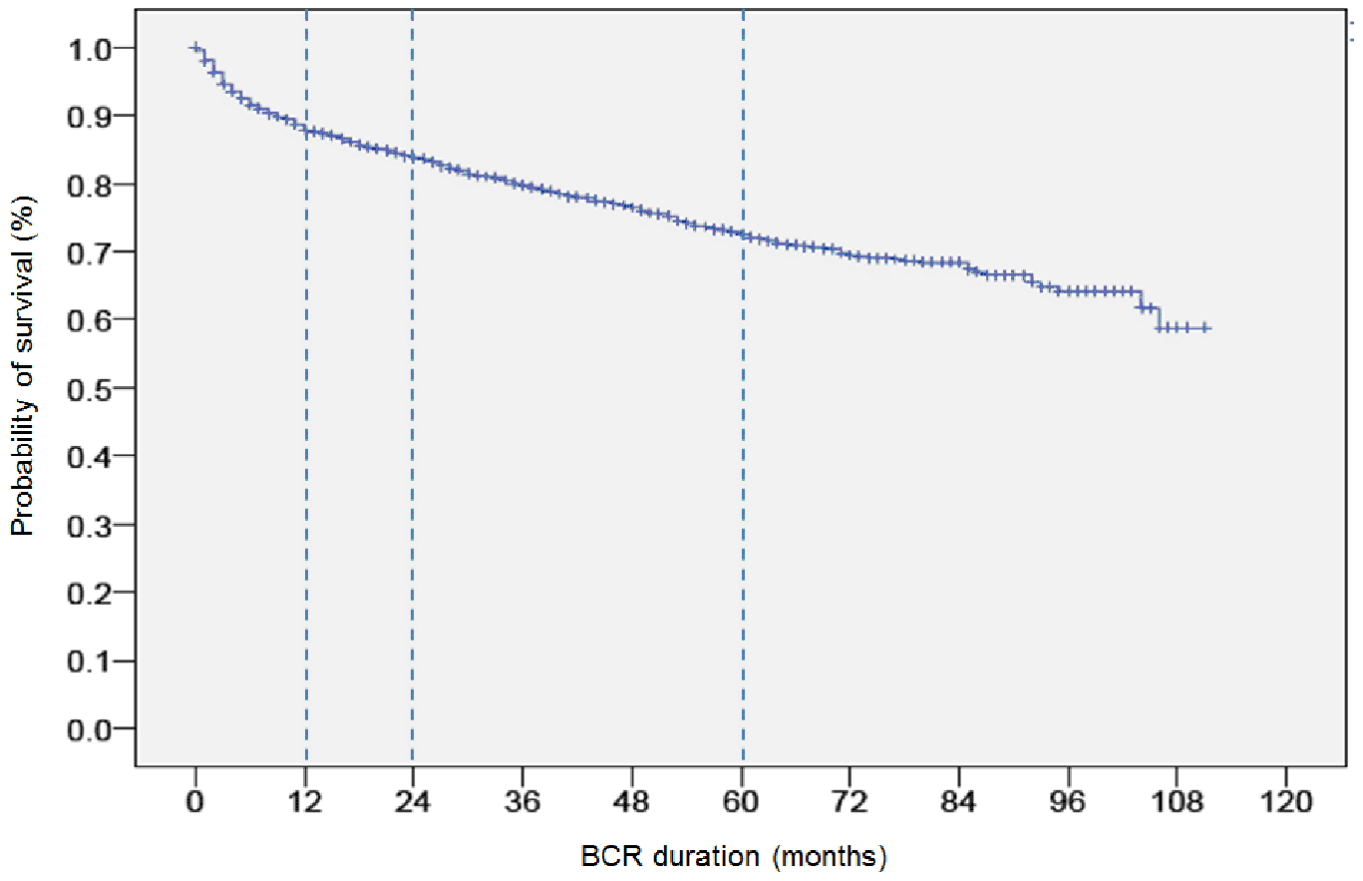
Biochemical recurrence (BCR)-free probability during follow-up after radical prostatectomy.

The clinical features of the patients are described in Table 1. The mean age was 65.9±6.6 (IQR: 62–71) at the time of RP. The mean preoperative serum PSA level was 11.6±12.2 ng/mL (IQR: 5.1–12.7 ng/mL). Of note, a large proportion of patients in this study had non-aggressive clinical features, including clinical T1c and T2a in 2,726 cases (95.4%), biopsy GS ≤6 in 1,394 cases (48.6%). In contrast, pathologic characteristics were turned to be more aggressive, including EPE in 990 cases (34.5%), SVI in 298 (10.4%), pathologic GS 7 (3+4) in 1,247 cases (43.5%), and GS 7 (4+3) in 547 cases (19.1%). The median follow-up was 47 months. BCR-free survival of the total patients is shown in Figure 1. The estimated BCR-free survival rates were 87.8% at 1 year, 83.8% at 2 year, and 72.5% at 5 year.

**Table 1:** The clinicopathologic characteristics of the patients.

Variables	Total number (%)
Age, years (Mean ± SD)	65.9±6.6 (IQR:62–71)
PSA, ng/ml	11.6±12.2 (IQR:5.1–12.7)
Clinical T stage (%)	
≤T1c	1,726 (60.2%)
T2a	1,009 (35.2%)
T2b	28 (1.0%)
T2c	88 (3.1%)
T3a	16 (0.6%)
Biopsy GS (%)	
≤6	1,394 (48.6%)
7 (3+4)	568 (19.8%)
7 (4+3)	332 (11.6%)
8	305 (10.6%)
≥9	146 (5.1%)
Missing	122 (4.3%)
EPE (%)	990 (34.5%)
SVI (%)	298 (10.4%)
LNM (%)	73 (2.5%)
SM+(all)	974 (34.0%)
SM+in T2	341/1,846 (18.5%)
Pathologic GS (%)	
≤6	781 (27.2%)
7 (3+4)	1247 (43.5%)
7 (4+3)	547 (19.1%)
8	91 (3.2%)
≥9	201 (7.0%)

Abbreviations: PSA, Prostate-Specific Antigen; GS, Gleason score; EPE, extra-prostatic extension; SVI, seminal vesicle invasion; LNM, lymph node metastasis; surgical margin, SM; HR.

Pre- and post-operative multivariable Cox PH regression models appear in Table 2. In the pre-operative model, preoperative PSA (hazard ratio [HR] 2.802, 95% confidence interval [CI] 2.177–3.608, *P*<0.001), PPC (HR 1.011, 95% CI 1.008–1.015, *P*<0.001), clinical T3a (versus T1c) (HR 2.281, 95% CI 1.197–4.345, *P* = 0.012) and biopsy GS (GS 7 to 10 versus GS 6, *P*<0.001) were independent predictive factors for BCR. Notably, all entered variables (preoperative PSA, EPE, SVI, LNM, SM and pathologic GS) were associated with BCR in the post-operative model. Among these prognostic factors, pathologic GS was the most significant predictor of disease recurrence, for example, the multivariable HR of GS 10 was 7.908 (95% CI 4.376–14.249, *P*<0.001) when compared to GS6.

**Table 2:** Pre- and post-operative Cox proportional hazards regression models.

	HR (95% CI)	*P* value
**Pre-operative models**		
Log PSA (ng/ml)	2.802 (2.177–3.608)	<0.001
PPC (% Positive core)	1.011 (1.008–1.015)	<0.001
Clinical T stage (vs. T1c)		0.074
T2a	1.145 (0.962–1.364)	0.127
T2b	1.001 (0.544–1.842)	0.998
T2c	1.297 (0.913–1.842)	0.147
T3a	2.281 (1.197–4.345)	0.012
Biopsy GS (vs. 6)		0.001
7 (3+4)	1.824 (1.402–2.375)	0.001
7 (4+3)	3.066 (2.347–4.007)	0.001
8	4.633 (3.568–6.016)	0.001
9	4.908 (3.599–6.693)	0.001
10	7.908 (4.376–14.249)	0.001
**Post-operative models**		
Log PSA (ng/ml)	1.384 (1.086–1.764)	0.009
EPE	1.854 (1.521–2.261)	<0.001
SVI	1.861 (1.517–2.285)	<0.001
LNM	1.470 (1.100–1.965)	0.009
SM+	1.930 (1.616–2.305)	<0.001
Pathologic GS (vs. 6)		<0.001
7 (3+4)	2.018 (1.478–2.756)	<0.001
7 (4+3)	4.201 (3.043–5.800)	<0.001
8	4.513 (2.945–6.916)	<0.001
≥9	6.325 (4.437–9.015)	<0.001

Abbreviations: PSA, Prostate-Specific Antigen; PPC, the proportion of positive biopsy cores; GS, Gleason score; EPE, extra-prostatic extension; SVI, seminal vesicle invasion; LNM, lymph node metastasis; surgical margin, SM; HR, hazards ratio; CI, confidence interval.

**Figure 2:**
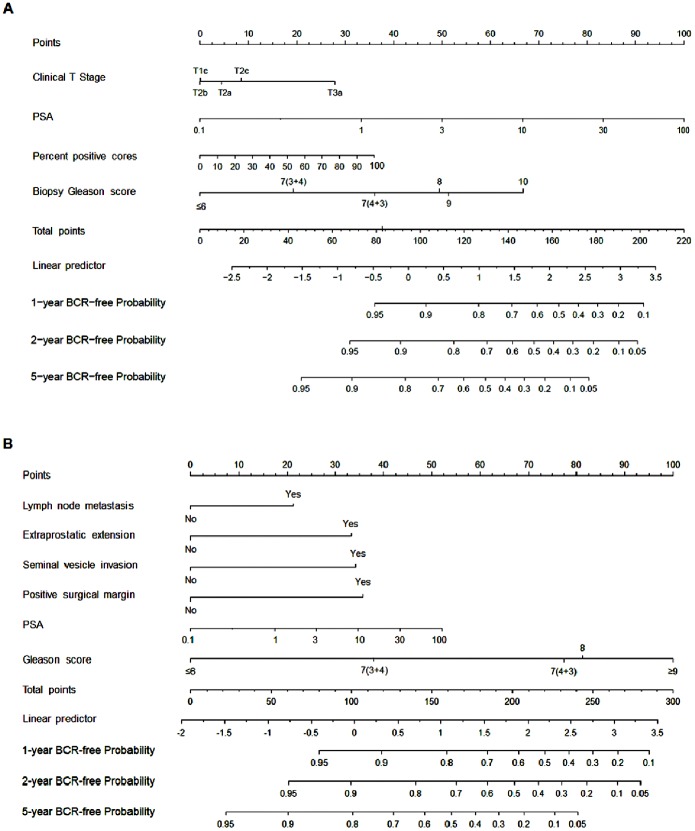
Pre- and post-operative nomograms predicting biochemical recurrence (BCR)-free probability at 1, 2, and 5 years after radical prostatectomy. Panel (A) represents pre-operative nomogram and panel (B) shows post-operative nomogram.

**Figure 3:**
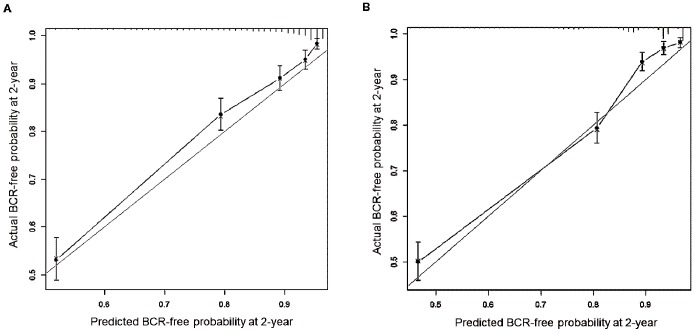
Calibration plots for pre- and post-operative nomograms predicting biochemical recurrence (BCR)-free probability on internal validation for 2 years after radical prostatectomy. Panel (A) and (B) represent calibration plots, respectively, for pre- and post-operative nomograms. Bootstrapping method was used for the internal validation of these nomograms. Grey line indicates the predictive performance of a perfect nomogram, and black line represents the predictive accuracy of our nomograms.

Based on these results, we established graphical pre- and post-operative nomograms predicting the probability of BCR after RP (Figure 2-A and 2-B). On internal validation, the predictive accuracy of pre- and post-operative nomograms was 0.792 and 0.821 of c-index, respectively. The calibration plot for pre- (Figure 3-A) and post-operative nomograms (Figure 3-B) showed good correspondence between predicted and actual probability of BCR at two years, indicating these nomograms were well calibrated.

## Discussion

Patients diagnosed with PCa are commonly involved in determining their therapeutic options based on the likelihood of oncologic control, complications and quality of life related to treatments, which can be predicted by validated statistical models [16]. Accurate prediction of individual risk of disease recurrence is essential in determining surveillance and adjuvant therapeutic strategy after local definitive therapy [8]. In this study, we developed pre- and postoperative nomograms to predict 1, 2, and 5 years freedom from BCR after RP based on a large Korean clinical series cohort with localized PCa. We evaluated all clinicopathologic data with Cox proportional hazards and bootstrapping methods that are current standard methods to develop a new nomogram in this field [17]. A nomogram should be calibrated with internal and external validation as gold standard method [18]. To evaluate our nomograms’ accuracy, we used a bootstrapping (resampling) that is a widely used statistical method for internal validation [19]. The c-index of pre- and post-operative nomograms was 0.792 and 0.821, validated by calibration plots. These results indicate that our nomograms are highly accurate and discriminate well, similar to well-known Western nomograms, and can be useful to predict BCR probability in Korean patients with clinically localized PCa who underwent RP.

Kattan *et al.* first reported a pre-operative nomogram (c-index 0.79) for disease recurrence using Cox proportional hazards regression analysis based on the data of 983 men with clinically localized PCa treated with RP [20]. They also developed a post-operative nomogram (c-index 0.89) for BCR after RP, based on 996 men with PCa treated with RP [21]. Later, they extended the prediction to 10 years after RP using Cox regression analysis based on 1,978 patients’ data, and the c-index of these updated nomograms was 0.76 on internal and 0.79 on external validation [22]. Urologists frequently use these nomograms when counseling their patients, because these models are regarded as accurate and discriminating prediction tools for BCR after RP. Despite the good performance of these Western nomograms, it is less than optimal to apply these tools directly to Korean patients because of geographic and ethnic/racial variations and environmental differences in clinical practice [11], [23]. Asian men, particularly Korean men, have a lower incidence of PCa, and more aggressive types of PCa compared to Western populations [24]–[26]. Similarly, approximately 45% of patients on TRUS biopsy and 75% of patients on pathologic examination of specimen had GS ≥7 in this study, whereas 30% of cases on biopsy and 45% of cases on pathology were observed with GS ≥7 in Western population-based pre- and post-operative nomograms [22]. In addition, PCa in Korea is not detected as early as in Western countries because PCa screening is not as commonly performed [27]. These discrepancies can undermine the accuracy of Western nomograms when they are recommended for Korean patients with PCa. Indeed, the updated Kattan nomogram showed a relatively lower c-index (0.76) with a poorly fitting calibration plot in Korean patients of PCa, while a higher c-index (0.81) was observed in a Western population [28]. The Seoul National University prostate cancer (SNU PC) stage calculator provided more accurate performance to predict several pathologic factors (organ-confined disease, EPE, SVI and LNM) than the 2013 Partin tables in a Korean multicenter cohort [12]. Furthermore, decision curve analyses, suggested as a novel approach to evaluate prediction models [29], also showed higher net benefits of the SNU PC stage calculator compared to 2013 Partin tables [12]. Therefore, different pathologic features and clinical environments should be considered when developing suitable ethnic- or nation-specific predictive models to overcome these limitations.

Cho *et al*. recently reported a post-operative nomogram predicting BCR-free survival, based on 723 Korean men with clinically localized PCa who underwent RP [28]. When they performed head-to-head comparison with the updated Kattan nomogram, their model gave superior predictions of BCR-free survival. Interestingly, they showed that preoperative PSA level was the strongest predictive factor on multivariable analysis. However, GS was the key independent predictor of disease recurrence in our post-operative nomogram. This result is consistent with current principals of considering GS the most significant predictor of disease recurrence among the three standard prognostic factors (GS, preoperative PSA and pathologic T stage) [30], [31]. Because the nomogram developed by Cho *et al*. was based on data collected from nineteen institutions, inter-individual variability for pathologic Gleason score interpretation can occur among pathologists. In contrast, we organized Seoul National University Uro-Oncology Group, and two experienced genitourinary pathologists communicated with each other periodically. That difference improves the quality of pathologic interpretation in this study, raising the reliability of our nomograms. Another important distinction of our prediction models is that we included LNM on multivariate analysis, now widely accepted as a significant risk factor for disease recurrence [31], while Cho *et al*. did not integrate LNM on their Korean nomogram.

There are several limitations to our pre- and post-operative nomograms. First, we collected and analyzed the data using retrospective methodology. Second, the five year time period may not be a definitive endpoint for predicting BCR because clinically significant prostate cancer can recur after five years of treatment [32]. We should extend the prediction of our models out to more than 10 years after RP. However, the majority of BCR are detected in the first three years after RP, and the patients with a shorter duration to BCR (3 years or less) are at a high risk of disease-specific death [33]. Thus, our nomograms predicting BCR can be useful to identify such high risk patients of disease recurrence after RP. Finally, we developed and validated these nomograms using patients treated at branch academic centers using a single practical set-up. To further determine the generalizability of these nomograms, external validation will be required by using populations of other academic hospitals or community based centers.

## Conclusions

In sum, we established pre- and post-operative nomograms that predict BCR-free probability with high accuracy and reliability after RP in a large Korean cohort with clinically localized PCa. These nomograms will be integrated into the mobile application-based calculator (SNUH Prostate Cancer Calculator), and thereby can be easily used to identify patients at high risk of disease recurrence who will benefit from adjuvant or salvage therapy.

## Supporting Information

Table S1 The clinicopathological data used for developing nomograms in the present study.
(XLSX)
